# The Differential and Dynamic Progression of Hepatic Inflammation and Immune Responses During Liver Fibrosis Induced by *Schistosoma japonicum* or Carbon Tetrachloride in Mice

**DOI:** 10.3389/fimmu.2020.570524

**Published:** 2020-10-07

**Authors:** Li-Jun Song, Xu-Ren Yin, Sha-Sha Mu, Jia-Huang Li, Hong Gao, Ying Zhang, Pan-Pan Dong, Cong-Jin Mei, Zi-Chun Hua

**Affiliations:** ^1^ School of Life Sciences and the State Key Laboratory of Pharmaceutical Biotechnology, Nanjing University, Nanjing, China; ^2^ National Health Commission Key Laboratory of Parasitic Disease Control and Prevention, Jiangsu Provincial Key Laboratory on Parasite and Vector Control Technology, Jiangsu Institute of Parasitic Diseases, Wuxi, China; ^3^ Public Health Research Center, Jiangnan University, Wuxi, China; ^4^ School of Biopharmacy, China Pharmaceutical University, Nanjing, China; ^5^ Jiangsu TargetPharma Laboratories Inc., Changzhou High-Tech Research Institute of Nanjing University, Changzhou, China; ^6^ Department of Pathology, Nanjing Drum Tower Hospital, The Affiliated Hospital of Nanjing University Medical School, Nanjing, China

**Keywords:** liver fibrosis, *Schistosoma japonicum*, carbon tetrachloride, hepatic inflammation, immune response, dynamic progression, differential progression

## Abstract

Liver fibrosis can result from various causes and could progress to cirrhosis and cancer; however, there are no effective treatments due to that its molecular mechanism is unclear. liver fibrosis model made by *Schistosoma japonicum* (*S. japonicum*) infection or Carbon tetrachloride (CCl_4_) intraperitoneal injection is a conventional model used in liver fibrosis-related studies for mechanism or pharmaceutical research purposes. But the differences in the pathological progression, immune responses and the underlying mechanism between the two liver fibrosis model have not been carefully compared and characterized, which hinders us from correctly understanding and making better use of the two models. In the present study, the pathological changes to the liver, and the cytokines, inflammatory factors, macrophages, and lymphocytes subsets involved were analyzed in the liver fibrosis model of *S. japonicum* infection or CCl_4_ intraperitoneal injection. Additionally, the pathological progression, immune responses and the underlying injury mechanism in these two models were compared and characterized. The results showed that the changing trend of interleukin-13 (IL-13), transforming growth factor beta (TGF-β), inflammatory factors, and M1, M2 macrophages, were consistent with the development trend of fibrosis regardless of whether liver fibrosis was caused by *S. japonicum* or CCl_4_. For lymphocyte subsets, the proportions of CD3^+^ T cells and CD4^+^ T cells decreased gradually, while proportion of CD8^+^ T cells peaked at 6 weeks in mice infected with *S. japonicum* and at 12 weeks in mice injected with CCl_4_. With prolonged *S*. *japonicum* infection time, Th1 (CD4^+^IFN-γ^+^) immunity converted to Th2 (CD4^+^IL-4^+^)/Th17 (CD4^+^IL-17^+^) with weaker regulatory T cell (Treg) (CD4^+^CD25^+^FOXP3^+^) immunity. However, in liver fibrosis caused by CCl_4_, Th1 cells occupied the dominant position, while proportions of Th2, Th17, and Treg cells decreased gradually. In conclusion, liver fibrosis was a complex pathological process that was regulated by a series of cytokines and immune cells. The pathological progressions and immune responses to *S. japonicum* or CCl_4_ induced liver fibrosis were different, possibly because of their different injury mechanisms. The appropriate animal model should be selected according to the needs of different experiments and the pathogenic factors of liver fibrosis in the study.

## Introduction

Liver fibrosis caused by, for example, metabolic diseases, virus infection, and drug toxicity, result in liver injury and inflammation. These factors stimulate the quiescent hepatic stellate cells (HSCs), which are activated and differentiate into myofibroblasts, secreting a large amount of extracellular matrix (ECM) components, such as collagen, glycoprotein, and proteoglycan. Meanwhile, myofibroblast tissue metalloproteinase inhibitors (TIMPs) secreted by cells inhibit the activity of matrix metalloproteinases (MMPs) in degrading the ECM, resulting in abnormal deposition of fibrous connective tissue in the liver and the formation of liver fibrosis (1, 2). The exact molecular mechanism of liver fibrosis is still unclear (3). Additionally, liver fibrosis could progress to cirrhosis and cancer, which are detrimental to health and may cause death (3). Previous experimental models have been confirmed that liver fibrosis could be reversed by increasing collagenolytic activity (4) or by removing activated HSCs *via* apoptosis (5). However, there are no effective drug treatments in the clinic. Therefore, it is very important to study the pathological characteristics and possible regulatory mechanisms of liver fibrosis to search for novel treatments.

*In vivo* models are indispensable tools to study molecular mechanisms. Currently, there are five categories of *in vivo* models for liver fibrosis: Chemical, dietary, surgical, genetically modified, and infection (6). Ethanol and carbon tetrachloride (CCl_4_) (7) in the chemical category, a high-fat diet (8) in the dietary category, and HBV infection (9) have been used widely to induce liver fibrosis in animal experiments. Infection with *schistosoma* parasites (10) is also a popular model of liver fibrosis.

liver fibrosis model made by *S. japonicum* infection or CCl_4_ intraperitoneal injection is a conventional models used in liver fibrosis-related studies for mechanism or pharmaceutical research purposes, for example, in the recent five years since June, 2015, there are over 95 papers using liver fibrosis model by *S. japonicum* infection and about 775 papers using liver fibrosis model by CCl_4_ injection. In several studies, both *S. japonicum* infection and CCl_4_ intraperitoneal injection models were used. Similar results were obtained in the two models (11–13). The difference between the two models was also reported, such as the expression of protein Septin4 (14). But the similarities and differences between the two liver fibrosis models have not been carefully compared and characterized. The comparisons between the two liver fibrosis models are helpful to understand the possible molecular mechanism and make better use of the two models.

Dynamic progressions of pathological changes, the cytokines and lymphocytes subsets in mice infected by *S. japonicum* have been reported in several reports with various results (15–18), while those in CCl_4_ induced liver fibrosis were rarely reported or detected at different time point (19, 20). It was difficulty to make comparisons between the two models from the previous literatures. In the present study, the dynamic progressions of pathological changes, the cytokines, inflammatory factors, macrophages, lymphocytes subsets and underlying injury mechanism involved were compared and characterized between the liver fibrosis model of *S. japonicum* infection and CCl_4_ intraperitoneal injection at the same time point using the same evaluation indexes and methods.

## Materials and Methods

### Ethics and Biosecurity Statement

All animals were placed in the facility, provided food and water according to the Convention. The intraperitoneal injection, infection and euthanasia (carbon dioxide asphyxia) were carried out according to the Guidelines for feeding and using experimental animals of the Ministry of science and technology of the people’s Republic of China ((2006) No. 398). According to the guidelines, suffering was minimized.

All experimental operations were performed in accordance with the Laboratories - General requirements for biosafety of the people’s Republic of China (GB19489-2008).

### Animals and Parasites

*S. japonicum* cercariae (strain isolated in Jiangsu, China), hatched from infected *Oncomelania* *hupensis*, were provided by the Department of Snail Biology, Jiangsu Institute of Parasitic Diseases. Mice (C57BL/6), weighing 22–24 g, were purchased from the Shanghai Sub-Center of Experimental Animals, Chinese Academy of Sciences, and raised in the Department of Experimental Animals, Jiangsu Institute of Parasitic Diseases.

### Establishment of the Hepatic Fibrosis Model Using S. japonicum

**Figure 1:**
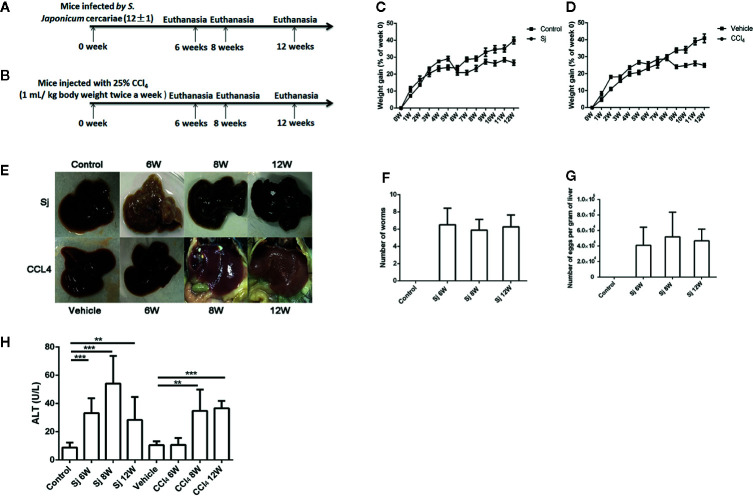
The schematic diagram, weight gain and serum ALT of mice infected with *S. japonicum* or injected with CCl_4_ intraperitoneally. **(A)** The schematic diagram of mice infected with *S. japonicum.* **(B)** The schematic diagram of mice injected with CCl_4_ intraperitoneally. **(C)** The weight gain of mice infected with *S. japonicum* (% of week 0). **(D)** Bodyweight gain of mice injected with CCl_4_ intraperitoneally (% of week 0). **(E)** Image of the morphology of liver in mice infected with *S. japonicum* and injected with CCl_4_ intraperitoneally. **(F)** The number of adult worm in mice infected with *S. japonicum* challenged with the cercariae (12 ± 1). **(G)** The number of eggs per gram of liver in mice infected with *S. japonicum* challenged with the cercariae (12 ± 1). **(H)** The serum ALT levels in mice infected with *S. japonicum* or injected with CCl_4_ intraperitoneally. ALT, alanine aminotransferase; SJ, *S. japonicum.* Vehicle, olive oil. Data represent the mean ± SE from three independent experiments. ^**^ *P* < 0.01, ^***^ *P* < 0.001.

The infected *Oncomelania hupensis* were placed in dechlorinated water and the cercariae spontaneously escaped from them under 25°C incubation. Normal female C57BL/6J mice, aged 6–8 weeks, 10 mice in each group of 6, 8, and 12 weeks, were challenged with the cercariae (12 ± 1) for 20 min (21). Blood was collected from the mouse’s eyeballs. Then the mice were euthanized at 6, 8, and 12 weeks after infection, and uninfected mice were used as a control group. The worms were perfused from portal veins and the number of worms was counted. The mouse livers were digested overnight with 5% potassium hydroxyl solution at 37°C and the number of eggs in the liver tissue was counted under an inverted phase contrast microscope. The number of eggs per gram of liver = the total number of eggs in the liver tissue/the liver weight. The mice were weighed weekly (**Figure 1A**).

### Establishment of the Hepatic Fibrosis Model Using CCl_4_

Normal female C57BL/6J mice, 6–8 weeks old, 6 mice in each group of 6, 8, and 12 weeks, were used. CCl_4_ (Shanghai Oulu Bio-Technology Co., Ltd (Shanghai, China)) was diluted with olive oil to obtain a 25% solution. Mice were injected intraperitoneally with 1 mL of 25% CCl_4_ per kg body weight twice a week for 6, 8, and 12 weeks, consecutively****(22). Blood was collected from the mouse’s eyeballs at 6, 8, and 12 weeks. Then the mice were euthanized and the livers were taken for assay. Mice receiving the control solvent olive oil were used as the vehicle group. The mice were weighed weekly (**Figure 1B**).

### Serum Collection From Mice and the Detection of Alanine Aminotransferase (ALT)

Blood was collected from the mouse’s eyeballs, stored overnight at 4°C, and then centrifuged at 1,000 g for 5 min. The upper layer (serum) was removed and stored at −80°C. The ALT level in the serum was detected using Alanine aminotransferase Assay Kit from the Nanjing Jiancheng Biotechnology Co., Ltd (Nanjing, China).

### The Colorimetric Hydroxyproline Assay in the Liver Tissue of Mice

The hydroxyproline levels in the mouse liver (30–100 mg) were detected using Hydroxyproline Assay Kit from Nanjing Jiancheng Biotechnology Co., Ltd.

### Masson Staining of Mouse Liver Tissue

The right anterior lobe of the mouse liver was excised and immersed in 4% polyformaldehyde for fixation. The tissue was dehydrated in 70%, 80%, 95%, and 100% ethanol in turn. The dehydrated liver tissue was transparent in ethanol/xylene. After tissue immersion and embedding, wax blocks were continuously sectioned using a microtome at a thickness of 4 μm, and then attached to a slide. A Masson staining kit from Nanjing Jiancheng Biotechnology Co., Ltd. was used to stain the sections. Each section was randomly acquired from 6–8 visual fields under a microscope. Images were taken and Masson staining positive areas were analyzed using Image-Pro software (Media Cybernetics, Rockville, MD, USA).

### Fluorescence Quantitative PCR Reactions

Total RNA was extracted from the mouse liver using Trizol. Total RNA (500 ng) was subjected to reverse transcription to produce cDNA using Transcriptor First Strand cDNA Synthesis kit (Roche, Basel, Switzerland), which was used as the template for fluorescence quantitative real-time PCR (qPCR) reactions to detect of cytokine mRNA levels using SYBR Green I Master (Roche, Basel, Switzerland) and LightCycler 480 instrument (Roche, Basel, Switzerland). The reaction procedure was as following: 95°C for 30 s; 40 cycles of 95°C for 5 s, 56°C for 10 s, 72°C for 15 s; 95°C for 15s, 60°C for 10s. 18S was used as an internal control, and the fold changes were quantified by the 2^-△△Ct^ method. The sequences of the primers are shown in **Supplementary Table 1** and all primer sequences were blasted in NCBI for ensuring their specificities.

### Flow Cytometry

Hepatic nonparenchymal cells were prepared as described previously with some modifications (23). The livers of 6–10 mouse were cut into small pieces and digested by 0.5 mg/ml collagenase and 4 U/ml DNase I in PBS at 37 °C for 30 min. The digested was filtered using 200-mesh and 400-mesh sieves, centrifuged for 5 min at 300 × *g* and terminated by adding phosphate-buffered saline (PBS). Hepatic parenchymal cells were removed after centrifugation at 30 × *g* for 5 min. The supernatant cells were centrifuged for 5 min at 300 × *g* in a new tube. The cells were then suspended in the D-Hanks solution. The suspension was slowly added into a centrifuge tube containing OptiPrep (40% v/v) to a final concentration of 20% (v/v) and overlaid with D-Hanks solution, centrifuged at 1500 × *g* 20 min. After centrifugation, the interface of cells was extracted, which comprised lymphocytes, macrophages, monocytes, HSCs cells and other cells, called nonparenchymal cells. The nonparenchymal cells (2 × 10^6^) were added with the following labeled antibodies: F4/80-phycoerythrin (PE) (Becton, Dickinson and Company, New Jersey, USA) CD16/32-FITC (Becton, Dickinson and Company, New Jersey, USA), CD206-allophycocyanin (APC) (Becton, Dickinson and Company, New Jersey, USA) (for M1, M2 macrophages); CD3-PE(Becton, Dickinson and Company, New Jersey, USA), CD4-FITC (Becton, Dickinson and Company, New Jersey, USA), CD8-APC (Becton, Dickinson and Company, New Jersey, USA) (for T cells). The proportions of macrophages and T cells in the samples were detected using flow cytometry. The nonparenchymal cells (2 x 10^6^) were cultured in 24-well plates. A cell stimulation cocktail was added into each well and the plates were placed at 37°C for 5 h in 5% CO_2_ incubator. The supernatant was discarded. Then, fixation/permeabilization solution was added and incubated for 20 min at 4°C. The cells were washed twice with 1 x perm/wash, and CD4-FITC, CD25-APC, forkhead box P3 (FOXP3)-PE, IL-4-PE, interferon gamma (IFN-γ)-APC, IL-17A-PE antibodies were added. The cells in the samples were then detected using flow cytometry.

### Statistical Analysis

The percentage of weight gain, the ALT levels in blood, the hydroxyproline levels in liver tissue, the mRNA levels of inflammatory factors, and the proportion of cells in liver tissue detected by flow cytometry between the two groups were tested using a t-test. SPSS 13.0 (IBM Corp., Armonk. NY, USA) was used for the statistical analyses. Differences between mean values were considered significant at *P* < 0.05.

## Results

### The Weight and Serum ALT in Mice Infected with S. japonicum or Injected Intraperitoneally With CCl_4_

The mortality of mice infected with *S. japonicum* was 30.0% at 9W post infection and 28.6% at 10W post infection. By contrast, the mortality of mice injected with CCl_4_ within 12 weeks was 0.0%. The weight gain (% of week 0) of mice infected with *S. japonicum* was significantly reduced (*P=*0.000) at 7 weeks compared with that of the control group (**Figure 1C**). The weight gain (% of week 0) of the mice in the CCl_4_ liver fibrosis model increased slowly with time but decreased at 9 weeks (**Figure 1D**). The liver surface of mice infected with *S. japonicum* had obvious egg nodules, and diffuse granular nodules on the liver surface of mice injected with CCl_4_ were seen (**Figure 1E**). The number of adult worms in mice infected by *S. japonicum* at 6, 8, 12 weeks was 6.5 ± 1.9, 5.9 ± 1.2, 6.3 ± 1.4, respectively (**Figure 1F**). The number of eggs per gram of liver in mice infected by *S. japonicum* at 6, 8, 12 weeks was 41068.1 ± 23,344.0, 51,838.46 ± 31,883.6, 46759.15 ± 15,105.8, respectively (**Figure 1G**). The level of ALT in the sera of mice infected with *S. japonicum* was 33.2 ± 10.6 U/L at 6 weeks, 54.2 ± 19.5 U/L at 8 weeks, and 28.4 ± 16.3 U/L at 12 weeks, which were all significantly higher than the 8.9 ± 3.4 U/L in the control group (*P*=0.000, 0.000, 0.002). At 6, 8, and 12 weeks, the levels of ALT in sera of mice intraperitoneally injected with CCl_4_ were 10.6 ± 4.9 U/L, 34.8 ± 15.0 U/L, and 36.7 ± 5.2 U/L, respectively. Compared with that in the control group, the levels at 8 and 12 weeks had statistical significance (*P*=0.003, 0.000) (**Figure 1H**). The level of ALT in mice infected with *S. japonicum* at 6 weeks was higher than that of mice injected with CCl_4_. The difference had statistical significance (*P*=0.000).

### The Intensity of Liver Fibrosis in Mice Infected With S. japonicum or Injected With CCl_4_ Intraperitoneally

**Figure 2:**
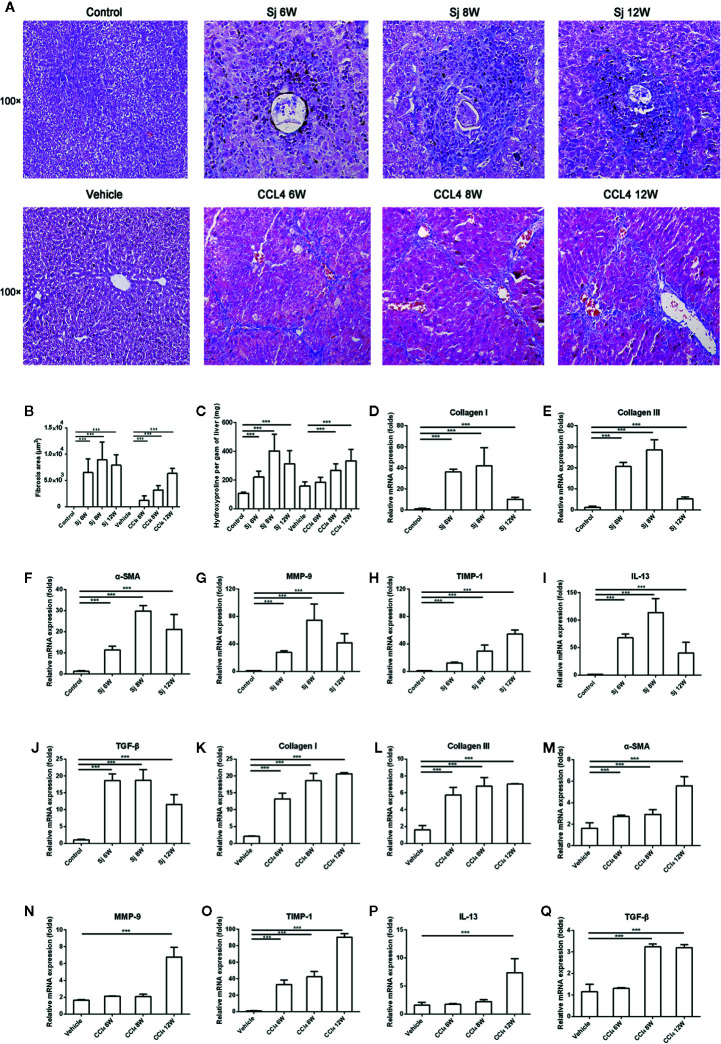
The intensity of liver fibrosis and cytokine levels in the liver of mice infected with *S. japonicum* or injected with CCl_4_ intraperitoneally. **(A)** Masson staining of the liver in mice. Original magnification 100×. **(B)**The fibrosis areas of the liver of mice infected with *S. japonicum* or injected with CCl_4_ intraperitoneally. **(C)** The hydroxyproline level in the mice liver. **(D–J)** The relative mRNA expression levels of collagen I, collagen III, α-SMA, MMP-9, TIMP-1, IL-13, and TGF-β in the liver of mice infected with *S. japonicum.* **(K–Q)** The relative mRNA expression levels of collagen I, collagen III, α-SMA, MMP-9, TIMP-1, IL-13, and TGF-β in the liver of mice injected with CCl_4_ intraperitoneally. SJ, *S. japonicum* Vehicle, olive oil. Data represent the mean ± SE from three independent experiments. ^***^ *P* < 0.001.

Mice infected with *S. japonicum* developed fibrosis in relation to the granulomas formed around the parasite’s eggs. At the end of the experiment (12 weeks), there was fibrosis around the granulomas and sometimes granulomas fused by the fibrosis (**Figure 2A**). At 6, 8, and 12 weeks, the fibrotic area of a single egg caused by *S. japonicum* was 6,517.3 ± 2,624.5 μm^2^, 8,941.4 ± 3,414.3 μm^2^, and 7,935.4 ± 2,001.8 μm^2^, respectively. The differences were statistically significant compared with that of the control group (*P*=0.000, 0.000, 0.000) (**Figure 2B**). The levels of hydroxyproline in the liver were 220.5 ± 42.2 mg/g, 403.4 ± 114.6 mg/g, and 313.7 ± 91.1 mg/g at 6, 8, and 12 weeks, respectively. The differences were statistically significant compared with that of the control group (108.7 ± 7.0 mg/g) (*P*=0.000, 0.000, 0.000) (**Figure 2C**). The fibrotic area of a single egg and the level of hydroxyproline in the liver caused by *S. japonicum* peaked at 8 weeks, as did the levels of mRNA encoding collagen I and III, and α-smooth muscle actin (α-SMA) in the liver (**Figures 2D–F**). The level of mRNA encoding MMP9 peaked at 8 weeks, but decreased at 12 weeks, while the mRNA levels of TIMP1 increased with time (**Figures 2G, H**). The mRNA expression levels of interleukin 13 (IL-13) and TGF-β (related to fibrosis) were the highest at 8 weeks and decreased at 12 weeks (**Figures 2I, J**).

In CCl_4_-treated mice, fibrosis developed from areas of necrosis mainly located in zone three of hepatic acini. At the end of the experiment, there was septal fibrosis with the formation of septa connecting the central veins to the portal spaces and connecting the portal spaces to each other. Pseudo lobules surrounded by collagen fibers were observed (**Figure 2A**). The areas of fibrosis caused by CCl_4_ were 1236.5 ± 842.6 μm^2^, 3203.5 ± 867.6 μm^2^, and 6352.5 ± 1008.2 μm^2^ at 6, 8, and 12 weeks, respectively, which were significantly different compared with that of the control group (*P*=0.000, 0.000, 0.000) (**Figure 2B**). The levels of hydroxyproline in liver were 184.9 ± 34.7 mg/g, 268.3 ± 44.4 mg/g, and 333.4 ± 80.0 mg/g at 6, 8, and 12 weeks, respectively (**Figure 2C**). The area of CCl_4_-induced fibrosis and the hydroxyproline in the liver reached the highest value at 12 weeks. The levels of mRNA encoding Collagen I and III, and α-SMA in the liver reached their highest values at 12 weeks (**Figures 2K–M**). MMP9 and TIMP1, the enzymes related to the degradation of fibrosis, showed their highest mRNA levels at 12 weeks (**Figures 2N, O**). The highest mRNA levels of IL-13 and TGF-β were found at 12 weeks (**Figures 2P, Q**).

The area of fibrosis in mice infected with *S. japonicum* at 6, 8, and 12 weeks was higher than that of mice injected with CCl_4_, respectively. The differences had statistical significances (*P*=0.000, 0.000, 0.000). The level of hydroxyproline in liver of mice infected with *S. japonicum* at 8 weeks was significantly higher than that of mice injected with CCl_4_ (*P*=0.008).

### The mRNA Levels of Inflammatory Factors in the Liver of Mice Infected With *S. japonicum* or Injected With CCl_4_ Intraperitoneally

**Figure 3:**
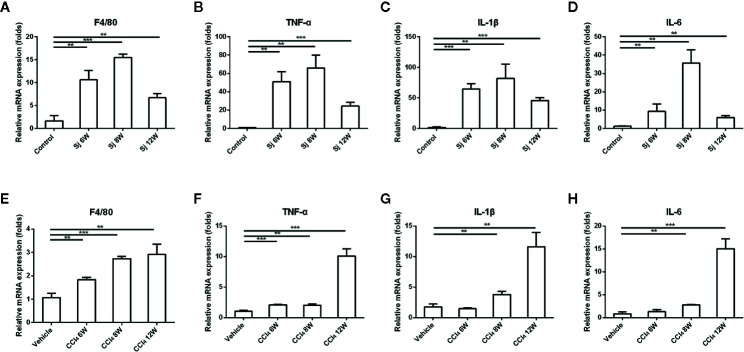
The mRNA levels of inflammatory factors in the liver of mice infected with *S. japonicum* or injected with CCl_4_ intraperitoneally. **(A–D)** The relative mRNA expression levels of F4/80, TNF-α, IL-1β and IL-6 in the liver of mice infected with *S. japonicum*. **(E–H)** The relative mRNA expression levels of F4/80, TNF-α, IL-1β, and IL-6 in the liver of mice injected with CCl_4_ intraperitoneally. SJ, *S. japonicum.* Vehicle, olive oil. Data represent the mean ± SE from three independent experiments. ^**^ *P* < 0.01, ^***^ *P* < 0.001.

The levels of mRNA encoding inflammatory factors epidermal growth factor-like module-containing mucin-like hormone receptor-like 1 (ERM1, also known as F4/80), interleukin 6 (IL-6), interleukin 1beta (IL-1β), and tumor necrosis factor alpha (TNF-α) in liver fibrosis caused by *S. japonicum* at 6, 8, and 12 weeks were significantly higher than those of the control (F4/80 compared with the control group at 6, 8, and 12 weeks, *P*=0.003, 0.000, 0.004; TNF-α compared with the control group at 6, 8, and 12 weeks, *P*=0.001, 0.001, 0.000; IL-1β compared with the control group at 6, 8, and 12 weeks, *P*=0.000, 0.004, 0.000; IL-6 compared with the control group at 6, 8, and 12 weeks, *P*=0.024, 0.001, 0.002), reaching their highest level at 8 weeks, and decreasing at 12 weeks. In the liver fibrosis caused by CCl_4_, the levels of mRNA encoding inflammatory factors F4/80, IL-6, IL-1β, and TNF-α reached a high value at 12 weeks. At 6, 8, and 12 weeks, the mRNA levels of F4/80 and TNF-α were statistically significantly higher than those in the control group (F4/80 compared with the vehicle group at 6, 8, and 12 weeks, *P*=0.003, 0.000, 0.003; TNF-α compared with the vehicle group at 6, 8, and 12 weeks, *P*=0.000, 0.002, 0.000), while IL-1β and IL-6 mRNA levels at 8 and 12 weeks were statistically significantly higher compared with those in the control group (IL-1β compared with the vehicle group at 8 and 12 weeks, *P*=0.008, 0.002; IL-6 compared with the vehicle group at 8 and 12 weeks, *P*=0.001, 0.000) (**Figure 3**).

### Flow Cytometry Analysis of Macrophages in the Liver of Mice Infected With S. japonicum or Injected Intraperitoneally With CCl_4_

**Figure 4:**
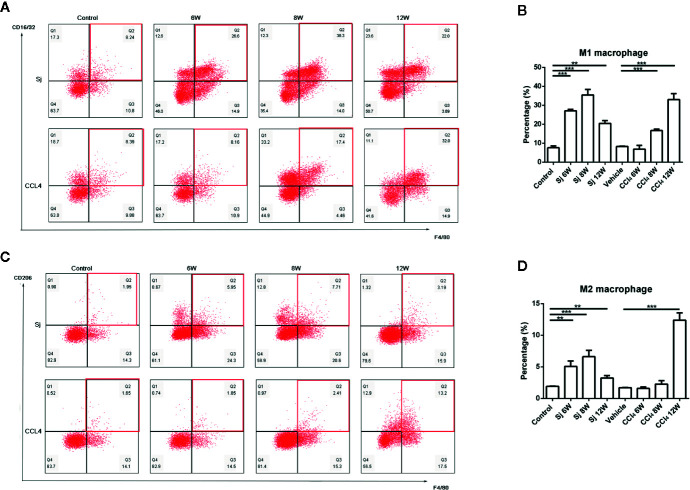
Flow cytometric analysis of macrophages in the liver of mice infected with *S. japonicum* or injected with CCl_4_ intraperitoneally. Scatter diagram of **(A)** M1 macrophages or **(C)** M2 macrophages in the mice liver. Statistical data analysis of **(B)** M1 macrophages or **(D)** M2 macrophages in the mice liver. SJ, *S. japonicum.* Vehicle, olive oil. Data represent the mean ± SE from three independent experiments. ^**^ *P* < 0.01, ^***^ *P* < 0.001.

The proportions of M1 macrophages in the liver of mice infected with S.* japonicum* were 27.13 ± 0.76%, 35.5 ± 2.90%, and 20.47 ± 1.50% at 6, 8, and 12 weeks, respectively, which were statistically significantly higher compared with that in the control group (7.60 ± 0.91%) (*P*=0.000, 0.000, 0.002) (**Figures 4A, B**). The proportions of M2 macrophages were 5.06 ± 0.89%, 6.61 ± 1.02%, and 3.23 ± 0.37% at 6, 8, and 12 weeks, respectively. The differences were statistically significant compared with that in the control group (1.91 ± 0.06%) (*P*=0.003, 0.001, 0.004) (**Figures 4C, D**).

The proportions of M1 macrophages in the liver of mice injected with CCl_4_ at 6, 8, and 12 weeks were 6.90 ± 1.98%, 16.7 ± 0.75%, and 33.03 ± 3.17% respectively. The differences between the proportions at 8 and 12 weeks and that of the control group (8.27 ± 0.16%) were statistically significant (*P*=0.000, 0.001) (**Figures 4A, B**). The proportions of M2 macrophages were 1.58 ± 0.23%, 2.26 ± 0.54% and 12.40 ± 1.14% at 6, 8, and 12 weeks, respectively. The proportion of M2 macrophage at 12 week was statistically significant compared with that in the control group (*P*=0.000) (1.69 ± 0.06%) (**Figures 4C, D**).

### Flow Cytometric Analysis of CD3/CD4/CD8^+^ Lymphocytes in the Liver of Mice Infected With S. japonicum or Injected Intraperitoneally With CCl_4_

**Figure 5:**
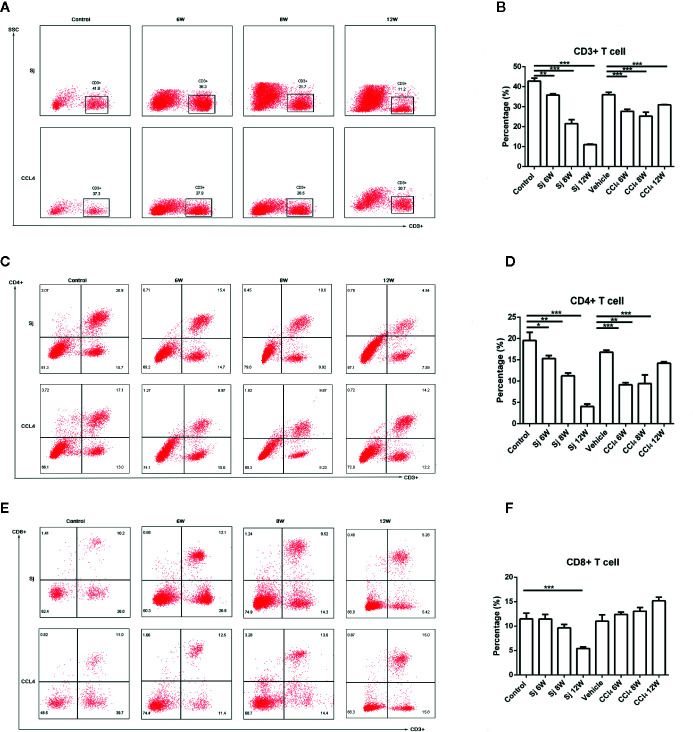
Flow cytometric analysis of CD3/CD4/CD8^+^ lymphocytes in the liver of mice infected with *S. japonicum* or intraperitoneally injected with CCl_4_. Scatter diagram of **(A)** CD3^+^ lymphocytes, **(C)** CD4^+^ lymphocytes or **(E)** CD8^+^ lymphocytes in the mice liver. Statistical data analysis of **(B)** CD3^+^ lymphocytes, **(D)** CD4^+^ lymphocytes or **(F)** CD8^+^ lymphocytes in the mice liver. SJ, *S. japonicum.* Vehicle, olive oil. Data represent the mean ± SE from three independent experiments. ^*^ *P* < 0.05, ^**^ *P* < 0.01, ^***^ *P* < 0.001.

For the *S. japonicum* model, the results showed that the percentages of CD3^+^ T cells in liver nonparenchymal cells were 35.80 ± 0.70%, 21.50 ± 2.00%, and 11.00 ± 0.26% at 6, 8, and 12 weeks, respectively, which were statistically significantly lower compared with that in the control group (42.85 ± 1.48%) (*P*=0.002, 0.001, 0.000) (**Figures 5A, B**). The proportions of CD4^+^ T cells in liver nonparenchymal cells were 15.30 ± 0.70%, 11.23 ± 0.68%, and 4.03 ± 0.61% at 6, 8, and 12 weeks, respectively, which were statistically significantly lower compared with that in the control group (19.55 ± 1.91%) (*P*=0.022, 0.002, 0.000) (**Figures 5C, D**). The proportions of CD8^+^ T cells in liver nonparenchymal cells were 11.45% ± 0.92%, 9.65 ± 0.70%, 5.43% ± 0.31% at 6, 8, and 12 weeks, respectively. The proportion of CD8^+^ T cells in the 12 weeks group was statistically significantly lower than that in the control group (11.47 ± 1.17%) (*P*=0.000) (**Figures 5E, F**).

The percentages of CD3^+^ T cells in the liver nonparenchymal cells of CCl_4_ liver fibrosis model were 27.67 ± 1.07%, 25.27 ± 1.94%, and 30.90 ± 0.20% at 6, 8, and 12 weeks, and the differences between the values at 6, 8 and 12 weeks and that of the control group (36.03 ± 1.20%) were statistically significant (*P*=0.000, 0.000, 0.001) (**Figures 5A, B**). The percentages of CD4^+^ T cells in the liver parenchyma cells were 9.12 ± 0.52%, 9.41 ± 2.05%, and 14.20% ± 0.36% at 6, 8, and 12 weeks, respectively, and the differences in the percentages at 6, 8, or 12 weeks compared with that in the control group (16.80 ± 0.45%) were statistically significant (*P*=0.000, 0.004, 0.001) (**Figures 5C, D**). The proportions of CD8^+^ T cells in liver nonparenchymal cells at 6, 8, and 12 weeks, respectively, were 12.36 ± 0.51%, 13.05 ± 0.78%, 15.20 ± 0.72%, and the proportion at 6, 8, 12 weeks had no statistical significance with that in the control group (11.00 ± 1.32%) (**Figures 5E, F**).

### Flow Cytometry for the Detection of Th1/Th2/Th17/Treg Cell in Mice Infected With S. japonicum or Injected Intraperitoneally With CCl_4_

**Figure 6:**
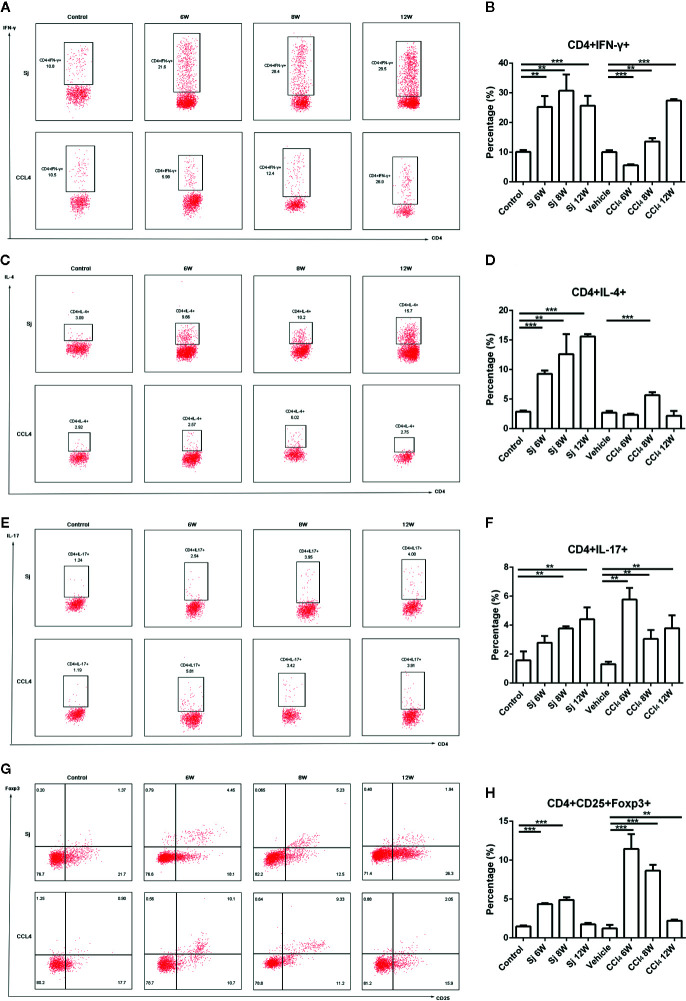
Flow cytometric for the detection of Th1/Th2/Th17/Treg cells in mice infected with *S. japonicum* or intraperitoneally injected with CCl_4_. Scatter diagram of **(A)** Th1 cells (CD4^+^IFN-γ^+^), **(C)** Th2 cells (CD4^+^IL-4^+^), **(E)** Th17 cells (CD4^+^IL-17^+^) or **(G)** Treg cells (CD4^+^CD25^+^Foxp3^+^) in total CD4^+^ T cells from mice liver. Statistical data analysis of **(B)** Th1 cells (CD4^+^IFN-γ^+^), **(D)** Th2 cells (CD4^+^IL-4^+^), **(F)** Th17 cells (CD4^+^IL-17^+^), or **(H)** Treg cells (CD4^+^CD25^+^Foxp3^+^) in total CD4^+^ T cells from mice liver. SJ, *S. japonicum*; Vehicle, olive oil; Treg, regulatory T cell. Data represent the mean ± SE from three independent experiments. Cells were gated on the CD4^+^ population. ^**^ *P* < 0.01, ^***^ *P* < 0.001.

In the *S. japonicum* model, flow cytometry showed that the proportions of Th1 (CD4^+^ IFN-γ^+^) cells in liver nonparenchymal cells were 25.30 ± 3.65%, 30.70 ± 5.47%, and 25.67 ± 3.33% at 6, 8, and 12 weeks, respectively, which were statistically significantly different compared with that in the control group (10.13 ± 0.58%) (P=0.002, 0.003, 0.001) (**Figures 6A, B**). The proportions of Th2 (CD4^+^IL-4^+^) cells in liver nonparenchymal cells were 9.27 ± 0.56%, 12.60 ± 3.39%, and 15.60 ± 0.40% at 6, 8, and 12 weeks, respectively, which were statistically significantly higher compared with that in the control group (2.87 ± 0.20%) (*P*=0.000, 0.007, 0.000) (**Figures 6C, D**). The proportions of Th17 (CD4^+^IL-17^+^) cells in liver nonparenchyma cells were 2.79 ± 0.47%, 3.78 ± 0.15%, and 4.41 ± 0.81% at 6, 8 and 12 weeks, respectively. The proportions of Th17 cells at 8 or 12 weeks were statistically significantly higher than that in the control group (1.57 ± 0.62%) (*P*=0.004, 0.008) (**Figures 6E, F**). The proportions of Treg (CD4^+^CD25^+^Foxp3^+^) cells in liver nonparenchyma cells were 4.34 ± 0.11, 4.88% ± 0.34%, and 1.74 ± 0.19% at 6, 8, and 12 weeks, respectively, which were statistically significantly higher compared with that in the control group (1.47 ± 0.14%) (*P*=0.000, 0.000) (**Figures 6G, H**).

In the CCl_4_ liver fibrosis model, the proportions of Th1 (CD4^+^IFN-γ^+^) cells in liver nonparenchymal cells were 5.60 ± 0.37%, 13.63 ± 1.09%, and 27.40 ± 0.43% at 6, 8, and 12 weeks, respectively, which were statistically significantly different compared with that of the control group (10.06 ± 0.62%) (*P*=0.000, 0.008, 0.000) (**Figures 6A, B**). The proportions of Th2 (CD4^+^IL-4^+^) cells in liver non parenchymal cells were 2.32 ± 0.22%, 5.67 ± 0.49%, and 2.16 ± 0.84% at 6, 8, and 12 weeks, respectively, and the proportion at 8 weeks group was statistically significantly higher compared with that in the control group (2.70 ± 0.31%) (*P*=0.000) (**Figures 6C, D**). The proportions of Th17 (CD4^+^IL-17^+^) cells were 5.77 ± 0.80%, 3.05 ± 0.62%, and 3.78% ± 0.89% at 6, 8, and 12 weeks, respectively, which were statistically significantly higher compared with that of the control group (1.31 ± 0.17%) (*P*=0.007, 0.009, 0.010) (**Figures 6E, F**). The proportions of Treg (CD4^+^CD25^+^Foxp3^+^) cells were present at 11.45 ± 1.90%, 8.64 ± 0.75%, and 2.20 ± 0.16% at 6, 8, and 12 weeks, respectively. Compared with that of the control group (1.22 ± 0.45%), the differences were statistically significant (*P*=0.000, 0.000, 0.001) (**Figures 6G, H**).

## Discussion

The weight gain of mice slowed dramatically at 6 weeks post-schistosome infection, while decreased at 9 weeks in the CCl_4_ liver fibrosis model, which was not reported in the previous literatures. The weight loss indicated that the health status of the body had declined. The liver morphological differences of the two models were that egg nodules on the liver surface of mice infected with *S. japonicum*, while diffuse granular nodules on that of mice injected with CCl_4_. The discrepancy may be related to the pathogenesis. The cercariae of *S. japonicum* develop into adults at 5 weeks post-infection. Female and male worms pair and eggs are laid, deposited into the liver (2). Mature eggs of *S. japonicum* in the liver causing an inflammatory granuloma, characterized by egg nodule. CCl_4_ produces free radical in the hepatocytes, which attack membrane unsaturated lipids and damage hepatocytes (24). The necrotic hepatocytes releasing damage associated molecular patterns (DAMPs), induced chronic inflammation followed by fibrosis. The proliferative collagenous fiber retracted the capsule, highlighting the regenerated parenchyma, characterized by diffuse granular nodules (25).

As the data greatly depend on the infection number of *S. japonicum* or the dosage, the time and frequency of the injection of CCl_4_, the dynamic progressions of pathological changes, the cytokines, inflammatory factors, macrophages and lymphocytes subsets were compared in the two models of liver fibrosis.

ALT exists in the cytoplasm of hepatocytes and is released into the blood when they are damaged; therefore, ALT was assayed to reflect the damage to hepatocytes (26). Hydroxyproline levels correlate significantly with liver fibrosis (27) and its level could reflect the intensity of fibrosis. The results showed that ALT, hydroxyproline, collagen I and III, and α-SMA mRNA levels all increased at 6 weeks, peaked at 8 weeks, and decreased at 12 weeks in response to *S. japonicum* infection. The pathological results showed that a small amount of blue collagen appeared around the egg granuloma at 6 weeks, which peaked at 8 weeks, and then decreased at 12 weeks. The lesions observed at different times were similar to those described in the literature (18). MMP-9, the metalloenzyme involved in collagen degradation, also reached a peak at 8 weeks, and then decreased. However, TIMP-1, the inhibitor of metalloenzymes, increased with the time of infection. The results indicated that collagen synthesis was more active than degradation. The balance between MMP/TIMP has been shown to be critical for the turnover of ECM (28). The progressions of collagen I and III, α-SMA, hydroxyproline, fibrosis intensity were generally consistent with previous reports (15, 16). The levels of ALT in serum were lower than those reported maybe relevant with different assay kits and methods (29).

Unlike the *S. japonicum* infection, the ALT, hydroxyproline, collagen I and III, and α-SMA mRNA levels in the livers treated with CCl_4_ increased with time, reaching their highest values at 12 weeks, which were not reported previously. MMP-9 and TIMP-1 mRNA levels increased with time; however, the increase in TIMP-1 was greater than that of MMP-9, which indicated that collagen synthesis was more active than degradation, resulting in fibrosis. Masson staining showed blue collagen concentrated in the portal area at the beginning of the CCl_4_ administration, and the degree of fibrosis increased with extended treatment time. The lesions at different times of observation were similar with previous reports (27).

The levels of cytokines IL-13 and TGF-β were consistent with the development trend of liver fibrosis caused by *S. japonicum* or CCl_4_, suggesting that TGF-β and IL-13 were important factors that promoted the progress of liver fibrosis. The results were similar to those reported previously. TGF-β-Smads signaling pathway was confirmed that could boost the activation and release of collagen from stationary HSCs (30). Furthermore, anti-TGF-β approaches have been established and utilized for the treatment of experimental liver fibrosis (31). IL-13 was reported that could promote liver fibrosis by activating HSCs through the protein kinase C pathway (32). Until recently it was believed that IL-13 could directly upregulate genes involved in the fibrotic response, including collagen I, collagen III, MMP and TIMP-1 (33, 34).

The changes in the levels of mRNA encoding TNF-α, F4/80, IL-1β, and IL-6 also correlated with the trend of fibrosis in the *S. japonicum* model. They all increased at 6 weeks, peaked at 8 weeks, and decreased at 12 weeks. Similar results were obtained for these mRNAs in the CCl_4_ fibrosis model. The changes in the levels of mRNA encoding TNF-α, F4/80, IL-1β, and IL-6 were in parallel to the trend of fibrosis in the CCl_4_ model. These results suggested that inflammation played an important role in liver fibrosis, and that the development of inflammation accelerated the progress of liver fibrosis, which agreed with the results of previous studies. Pradere and colleagues (35) confirmed that TNF-α and IL-1β accelerated liver fibrosis by promoting the survival of activated HSCs *in vitro* and *in vivo*. Miura and colleagues (36) demonstrated that in mouse non-alcoholic steatohepatitis (NASH), the production of IL-1β by macrophages could activate HSCs. Inflammatory mediators including TNF-α, IL-1β, and IL-10 may influence the progression of liver disease (37, 38). TNF-α stimulated ROS production in hepatocytes sensitized to undergo apoptosis, which induced HSCs activated (39).

At the cellular level, macrophages and lymphoid T cells are involved in liver fibrosis (40). In response to ongoing injury, M1 macrophages propagate inflammation and the development of fibrosis ultimately. Depending on cytokines in the microenvironment, M2 macrophages are recruited or activated as a result of an M1-to-M2 phenotype switch. M2 macrophages promote cell proliferation and reduce apoptosis (41–43). Our results showed that regardless of whether fibrosis was caused by *S. japonicum* or CCl_4_, the changing trend of macrophages, including M1 and M2 macrophages, were consistent with the development trend of fibrosis. M1 and M2 macrophages in liver nonparenchymal cells both peaked at 8 week, but decreased quickly in 12 week post *S. japonicum* infection. However, Zhu and colleagues reported that the percentage of M1 macrophages in peritoneum significantly increased 3 weeks but decreased by 8 weeks post-infection. While the percentage of M2 macrophages started to increase at 8 weeks after infection (44). The macrophages were detected in different tissues may lead to the discrepancy with the Zhu’s study. More M1 and M2-phenotypic markers including nitric oxide synthase (iNOS) and arginase-1 (Arg-1) would be tested in the future study. In CCl_4_-induced fibrosis, M2 macrophages may have more key roles in the later stage, which was not reported previously. The results of the two models suggested that both M1 and M2 macrophages were involved at each stage of liver fibrosis. The polarization of M1 or M2 macrophages depends on the cytokines in the microenvironment (45).

In T cells, our results showed that the proportions of CD3^+^, CD4^+^ T cells decreased with time, which might be relevant to the immune escape of *S. japonicum via* T cell apoptosis, especially CD4^+^ T cells (46). However, CD8^+^ T cell proportion reached their highest value at 6 weeks in mice infected with *S. japonicum*, suggesting that CD8^+^ T cells were mainly involved in the granuloma formation. It was reported (47) that the proportion of CD8^+^ T cells increased after infection at the early stage in the mice infected *S. mansoni*, with the function of inhibiting immunopathology. CD8^+^ T cells might be essential in the reduction of granuloma *in vivo* and *in vitro* (48, 49). In liver fibrosis caused by CCl_4_, the change trend of CD3^+^, CD4^+^ T cells was similar to that in mice infected by *S. japonicum*. The proportions of CD3^+^ T cells and CD4^+^ T cells decreased gradually, while CD8^+^ T cell proportion reached their highest value at 12 weeks, which was not reported previously. Previous experimental models implied that CD4 T-helper lymphocytes, especially Th1/Th2 balance may activate HSCs *via* cytokine production (50). Th1/Th2/Th17/Treg subtypes in CD4 T-helper lymphocytes were detected in our study.

With prolonged *S. japonicum* infection time, proportion of Th1 cells in liver nonparenchymal cells producing IFN-γ peaked at 8 weeks, and then decreased. Th2 cells secreting IL-4 predominated and reached their highest value at 12 weeks. The balance was consistent with previous reports (51). Th17 cell proportion gradually increased to a high value at 12 weeks, while Treg cells reached their highest levels at 8 weeks, and then declined. Our results were agreed with previous reported that weaker Treg responses were observed in the liver (52). In fact, a decreased expression of liver homing markers was detected on Tregs accumulated in the blood of patients with severe fibrosis during schistosomiasis (53). Our results showed that the immune response of host liver changed from Th1 to Th2/Th17 with weaker Treg, indicating that the antigen of eggs preferentially induced the generation of Th17 and Th2 cells (17). However, in liver fibrosis caused by CCl_4_, Th1 cells occupied the dominant position, while Th2, Th17, and Treg cells proportions decreased gradually. The host’s immune response was mainly Th1 type, a state of hyperactivity in liver microenvironment, which was not reported previously.

The reasons for differences in pathological progression and immune response may be related to the injury mechanism of the two modeling methods. Mature eggs of *S. japonicum* in the liver could release SEA into surrounding tissues, treated by APCs and presented to helper T cells. T cells can produce a variety of lymphokines, causing an inflammatory granulomatous reaction characterized by aggregation of macrophages, lymphocytes, neutrophils, and eosinophils. At this stage, the immunoreaction of the host was mainly Th1 activity secreting IFN-γ, which promoted inflammation. To avoid the damage caused by excessive inflammation to the body, the immunoreaction transformed into a Th2 immunosuppression response secreting IL-4, IL-10, and IL-13, induced by egg antigens (2). In *S. japonicum* infection, it has been reported that Th17 may play an important role in the formation and growth of granulomas around the eggs produced by the adult worm (17). The Treg cells with anti-inflammatory and anti-fibrotic role accumulate in the blood but failed to be recruited to the liver during schistosomiasis (53). Th2 response creates granulomas around schistosome eggs surrounded by a collagen ECM deposited by activated HSCs that protects the surrounding hepatocytes and other parenchymal cells from toxic egg-produced antigens (54). A dominant role of Th2 rather than Th1 response mediators during the schistosomiasis liver fibrotic process has been reported (55, 56).

CCl_4_ produces free CCl_3_· and Cl· after activation of a cytochrome P450-dependent oxidase in the hepatocyte endoplasmic reticulum. The free radicals bind covalently with macromolecules in hepatocytes, which could attack membrane unsaturated lipids, trigger the production of reactive oxygen species and lipid peroxidation, and damage hepatocytes, leading to necrosis, chronic inflammation and HSC activation, the release of collagen, and advanced liver fibrosis (20, 24). With the prolongation of treatment, the damage caused by CCl_4_ to hepatocytes accumulated gradually, increasing inflammation and fibrosis. The host immune system mainly promoted a Th1 type immune response to inflammation. Mahrouf-Yorgov and colleagues (57) reported that 8-week-old mice administrated with CCl_4_ for 6 weeks expressed less Th2 cytokines, while 15-month-old mice oriented toward a Th2 response. The type immune response may be connected with ages. Innate immune responses with a few participation of cell-mediated immunity may contribute to the progression of CCl_4_-induced fibrosis (20).

Altogether, in the present study IL-13, TGF-β, inflammatory factors, macrophages, and T lymphocyte contributed to the progress of liver fibrosis induced by *S. japonicum* and CCl_4_, but the time course or subtype of their participation were different, especially distinct T lymphocytes subtypes involved in the two models. The dynamic changes of pathological intensity reflected the above differences. The peak of pathological intensity appeared simultaneously with that of IL-13, TGF-β, inflammatory factors and macrophages in the two models, respectively.

## Data Availability Statement 

All datasets presented in this study are included in the article/**Supplementary Material**.

## Ethics Statement

The animal study was reviewed and approved by the ethics committee of Jiangsu Institute of Parasitic Diseases.

## Author Contributions

L-JS designed and performed the study; managed, analyzed, and interpreted the data; and prepared the manuscript. X-RY, S-SM, and HG assisted in the design and study implementation and revised the manuscript. J-HL and YZ designed the study and facilitated and assisted the study implementation. P-PD and C-JM assisted in the design of the study and data analysis. Z-CH designed the study, supervised the study implementation, analyzed the data, and revised the manuscript. All authors contributed to the article and approved the submitted version.

## Funding

This work was supported by grants from the National Natural Science Foundation of China (grant number 81802035, 81630092); the Project of Invigorating Health Care through Science, Technology and Education; the Scientific Research Projects from Jiangsu Provincial Commission of Health and Family Planning (grant number H201635); and the Public Health Research Center at Jiangnan University (grant number JUPH201812).

## Conflict of Interest

J-HL and Z-CH were employed by Jiangsu TargetPharma Laboratories Inc.

The remaining authors declare that the research was conducted in the absence of any commercial or financial relationships that could be construed as a potential conflict of interest.

## Supplementary Material

Supplementary Table 1 Primers used for mRNA analysis.
