# Supplementary material for: The Differential and Dynamic Progression of Hepatic Inflammation and Immune Responses During Liver Fibrosis Induced by Schistosoma japonicum or Carbon Tetrachloride in Mice
Source: Front Immunol. 2020 Oct 7;11:570524. doi: 10.3389/fimmu.2020.570524 (PMC7575768; doi:10.3389/fimmu.2020.570524)
Supplement: Supplementary Table 1 — Primers used for mRNA analysis. [file Table_1.docx]

Supplementary Table 1 | Primers used for mRNA analysis

| Genes | Primer | Sequence(5’–3’ ) |
| --- | --- | --- |
| 18S | Forward primer | TGCACCACCAACTGCTTAGC |
|  | Reverse primer | GTGGTCATGAGCCCTTCCA |
| Collagen I | Forward primer | GCGAGTGCTGTGCTTTCTG |
|  | Reverse primer | TCCCTCGACTCCTACATCTTC |
| Collagen III | Forward primer | CCCAACCCAGAGATCCCATT |
|  | Reverse primer | GAAGCACAGGAGCAGGTGTAGA |
| α-SMA | Forward primer | TCAGCGCCTCCAGTTCCT |
|  | Reverse primer | AAAAAAAACCACGAGTAACAAATCAA |
| MMP-9 | Forward primer | GCTCATGTACCCGCTGTATAGCT |
|  | Reverse primer | CAGATACTGGATGCCGTCTATGTC |
| TIMP-1 | Forward primer | TGGGAAATGCCGCAGATATC |
|  | Reverse primer | TGGGACTTGTGGGCATATCC |
| IL-13 | Forward primer | CCTGGCTCTTGCTTGCCTT |
|  | Reverse primer | GGTCTTGTGTGATGTTGCTCA |
| TGF-β | Forward primer | CTTTAGGAAGGACCTGGGTT |
|  | Reverse primer | CAGGAGCGCACAATCATGTT |
| F4/80 | Forward primer | CCCCAGTGTCCTTACAGAGTG |
|  | Reverse primer | GTGCCCAGAGTGGATGTCT |
| TNF-α | Forward primer | CCCTCACACTCAGATCATCTTCT |
|  | Reverse primer | GCTACGACGTGGGCTACAG |
| IL-1β | Forward primer | ATGGCAACTGTTCCTGAACTCAACT |
|  | Reverse primer | CAGGACAGGTATAGATTCTTTCCTTT |
| IL-6 | Forward primer | TAGTCCTTCCTACCCCAATTTCC |
|  | Reverse primer | TTGGTCCTTAGCCACTCCTTC |
